# Gene Expression Meta-Analysis of Cerebellum Samples Supports the FKBP5 Gene-Environment Interaction Model for Schizophrenia

**DOI:** 10.3390/life11030190

**Published:** 2021-02-27

**Authors:** Libi Hertzberg, Ada H. Zohar, Assif Yitzhaky

**Affiliations:** 1 Department of Physics of Complex Systems, Weizmann Institute of Science, Rehovot 76100, Israel; assif.yitzhaky@weizmann.ac.il; 2 Shalvata Mental Health Center, Affiliated with the Sackler School of Medicine, Tel-Aviv University, Tel Aviv 69978, Israel; 3 Department of Behavioral Sciences, Ruppin Academic Center, Hefer Valley 40250, Israel; adaz@ruppin.ac.il

**Keywords:** gene expression, meta-analysis, schizophrenia, FKBP5, cerebellum

## Abstract

Background: One of the most studied molecular models of gene-environment interactions is that of FKBP5, which has been shown to interact with childhood adversity to increase the risk of psychiatric disorders, and has been implicated in schizophrenia. While the model predicts up-regulation of FKBP5, previous brain samples gene expression studies yielded inconsistent results. Methods: We performed a systematic gene expression meta-analysis of FKBP5 and NR3C1, a glucocorticoid receptor inhibited by FKBP5, in cerebellum samples of patients with schizophrenia. The gene expression databases GEO, SMRI and those of NIMH were searched, and out of six screened datasets, three were eligible for the meta-analysis (overall 69 with schizophrenia and 78 controls). Results: We detected up-regulation of FKBP5 and down-regulation of NR3C1 in schizophrenia, and a negative correlation between their expression patterns. Correlation analysis suggested that the detected differential expression did not result from potential confounding factors. Conclusions: Our results give significant support to the FKBP5 gene-environment interaction model for schizophrenia, which provides a molecular mechanism by which childhood adversity is involved in the development of the disorder. To explore FKBP5’s potential as a therapeutic target, a mapping of its differential expression patterns in different brain regions of schizophrenia patients is needed.

## 1. Introduction

Childhood trauma has been shown to be associated with the development of schizophrenia [1,2]. While the genetic factor is estimated at around 80% [3], the effects of environmental factors are thought to be moderated by genetic variation [1]. In recent years, researchers have made significant progress in understanding the molecular mechanisms that underlie gene-environment interactions [4]. One of the most studied molecular models is that of FKBP5, which encodes the FK506 binding protein 51 (FKBP51). FKBP51 is a heat shock protein 90—associated co-chaperone that regulates stress responsiveness. When bound to the complex of glucocorticoid receptor (GR), the primary receptor responsive to stress-induced cortisol secretion, FKBP51 inhibits GR translocation into the nucleus, where it acts as a transcription factor [5].

Research by [6] presents a model for the interaction of FKBP5 and childhood adversity to increase the risk of psychiatric disorders: FKBP5 contains glucocorticoid response elements (GRE) that serve as enhancer motifs, where the GR can bind to and quickly induce FKBP5 gene expression. As FKBP5 inhibits GR signaling, this forms a short negative feedback loop for GR activation [7]. Adverse life experiences are associated with reduced DNA methylation at GREs, which leads to an increased transcriptional responsiveness of FKBP5 to future stressors. This effect is stronger in carriers of an intronic FKBP5 variant (rs1360780), which is associated with an enhanced GR-induced FKBP5 transcription [8]. By increasing FKBP5 transcriptional responsiveness, the variant also changes the negative feedback loop, leading to prolonged cortisol release in response to stress [9]. This in turn can further reduce DNA methylation in GREs and disinhibit FKBP5 transcription even more [10]. FKBP5 up-regulation has potential molecular, cellular, and brain structure consequences [6]. For example, elevated FKBP5 expression is associated with dendritic spine density changes in the orbitofrontal cortex of subjects with posttraumatic stress disorder (PTSD) [11]. These cortical processes precipitate early adversity-associated changes in brain structure and function and increase the risk for the development of a range of psychiatric disorders. There is evidence from preclinical studies that increased FKBP5 expression in specific brain regions is associated with risk-promoting sleep architecture and with increased stress responsiveness and anxiety [12,13,14,15], all of which are associated with increased vulnerability to psychiatric disorders.

Support for the FKBP5 gene-environment interaction model in schizophrenia comes from evidence for: (a) elevated cortisol levels in schizophrenia [16]; (b) impaired negative feedback observed in schizophrenia, leading to an excessive and prolonged stress response [17]; (c) reduced GR expression in hippocampus, frontal cortex, inferior temporal cortex [18] and amygdala samples of individuals with schizophrenia [19]. GR is known to be down-regulated by hypercortisolemia [20]. NR3C1, the GR encoding gene, is not associated with schizophrenia (nor with major depressive or bipolar disorders) as reported in recent genome-wide association studies (GWAS) (https://data.broadinstitute.org/mpg/ricopili/; accessed at 22 February 2021). However, NR3C1 methylation in blood samples was reported to be associated with schizophrenia, in females, supporting its role in the pathophysiology of schizophrenia [21]. As for FKBP5 itself, recent GWAS do not find an association with schizophrenia, nor with major depressive or bipolar disorders (https://data.broadinstitute.org/mpg/ricopili/; accessed at 22 February 2021). However, when early life stress interactions were taken into account, increased risk for psychosis was detected in two studies [22,23].

Several studies have explored FKBP5 differential expression patterns in post mortem brain samples of individuals with schizophrenia (summarized in Table 1). In [24], FKBP5 was found to be up-regulated in the hippocampus of patients with schizophrenia, while no differential expression was detected in the orbitofrontal cortex. In [25], concordantly down- and up-regulated genes across five major psychiatric disorders were identified. FKBP5 was detected among the top up-regulated genes across the five disorders, and it was most significantly up-regulated in schizophrenia ([25]; Figure S4). Interestingly, in a meta-analysis that included 196 brain samples of patients with schizophrenia and 194 controls, where 108 out of the 196 overlap the 159 schizophrenia samples used in [25] (listed in Table 1), no differential expression of FKBP5 was detected. Using multiple linear regression analysis, it was found that brain region was an influential factor on FKBP5 expression (*p*-value < 0.07) [26], suggesting that different brain regions should be analyzed separately. In an additional large sample group of the CommonMind Consortium (CMC; 258 schizophrenia vs. 279 control Dorso-lateral prefronal cortex (DLPFC) samples [27]), FKBP5 was not differentially expressed (*p*-value = 0.55; CMC FKBP5 differential expression was explored through the SZDB2.0 database [28,29]; see Figure S1). Thus, while there is evidence for up-regulation of FKBP5 in post mortem brain samples of individuals with schizophrenia, the results are inconsistent and the differential expression pattern might vary in different brain regions.

The common inconsistencies between the results of gene expression in schizophrenia studies [31] may be due to schizophrenia’s high heterogeneity [32] and to the differences in expression patterns across different brain regions [33]. Another limitation is the cellular complexity of the brain tissue and the fact that brain samples are usually composed of a mixture of different cell types. This can further increase the heterogeneity of the samples and might cause dilution, hence false negative results, of authentic gene expression changes that occur in a subpopulation of cells. One way to deal with these limitations is to integrate the results of independent datasets and analyze differential expression in each brain region separately. In addition to the enhanced statistical power provided by a meta-analysis, the fact that the analysis includes samples from distinct populations that were processed using different platforms notably strengthens the robustness and validity of the results.

We conducted a systematic gene expression meta-analysis of FKBP5 and NR3C1 in cerebellum samples of individuals with schizophrenia vs. healthy controls. We followed the PRISMA (Preferred Reporting Items for Systematic reviews and Meta-Analyses) guidelines, in order to minimize potential biases and to further increase the results’ validity [34]. Three publicly available gene expression datasets met the inclusion criteria. Our decision to focus on the cerebellum was based on the evidence for its involvement in schizophrenia [35,36] and the relatively high expression of FKBP5 in the cerebellum (http://mouse.brain-map.org/; accessed at 22 February 2021) [37]. In addition, the manageable number of relevant gene expression datasets made it feasible to conduct a systematic meta-analysis that follows accepted guidelines.

To deal with the differences in the platforms used and with the inherent heterogeneity of the patients, we used the Effect size combination with Random Effect Modeling, which takes both the direction and extent of differential gene expression into account, to create biologically consistent results [38]. To measure the potential effect of possible confounding factors, we computed the correlation between genes’ expression levels and several available such factors. In addition, in order to explore a potential role of FKBP5 and NR3C1 as biomarkers, we performed differential expression analysis of the two genes using a blood samples cohort of 13 individuals with schizophrenia and 8 controls.

## 2. Results

### 2.1. Selection of Eligible Gene Expression Datasets

**Figure 1:**
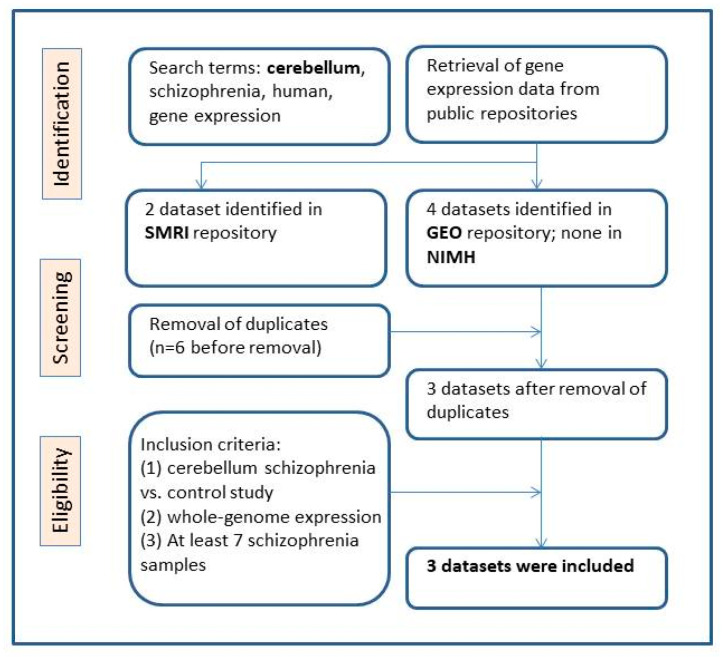
Workflow of meta-analysis.

Three gene expression datasets of post mortem cerebellum samples of individuals with schizophrenia versus controls met the inclusion criteria (Figure 1). Table 2 and the Supplementary Information provide descriptive information of the datasets.

### 2.2. The Cerebellum Gene Expression Datasets are Comparable

**Figure 2:**
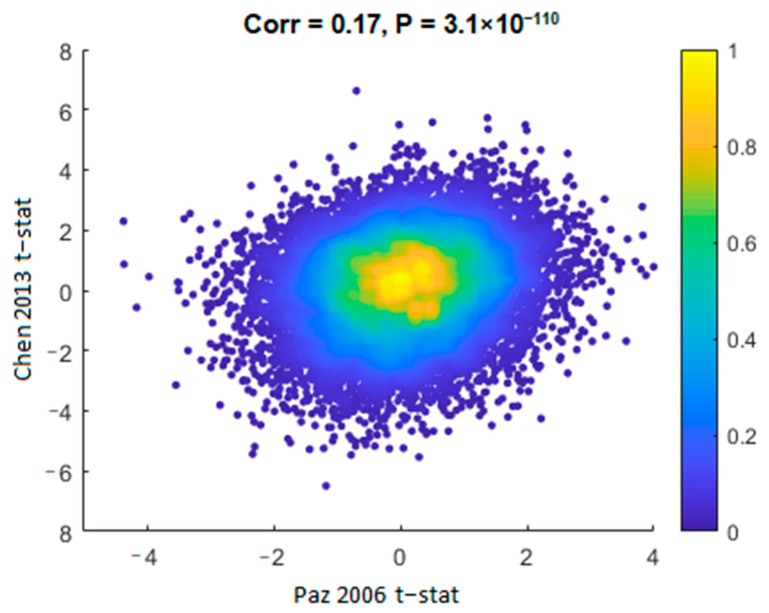
Binned density scatter plot comparing the t-statistics for schizophrenia versus controls differential expression between the Chen 2013 [40] and Paz 2006 [39] datasets; Pearson correlation between the statistics is 0.17 (*p* = 3.1×10^−110^). The colorbar represents the density in each cell, calculated by Voronoi procedure [38] and normalized to values between 0 (minimal density) and 1 (maximal density).

Although composed of distinct populations and processed using different platforms, we measured a significant positive correlation of the t-statistics (schizophrenia vs. control) between the Chen 2013 [40] and the Paz 2006 [39] datasets in all the genes common to both datasets (Corr. = 0.17, *p* = 3.1 × 10^−110^; Figure 2). A similar correlation was observed between Chen 2013 [40] and Stanley #6 datasets (Corr. = 0.14, *p* = 7.8 × 10^−39^). This measure avoids the issue of differences in statistical power due to different sample sizes and assesses the overall similarity or difference in gene expression alterations in a threshold-free manner.

It should be noted that there was a significant negative correlation between the Paz 2006 [39] and the Stanley #6 datasets (Corr. =−0.02, *p* = 0.03). However, as only one of the three pairwise comparisons had a negative correlation, and as it was much less significant than the positive correlations, we conclude that the three gene expression datasets are comparable, in terms of differential gene expression (schizophrenia vs. controls).

### 2.3. FKBP5 is Up-regulated and NR3C1, Encoding GR, is Down-regulated in Cerebellum Samples of Individuals with Schizophrenia

**Figure 3:**
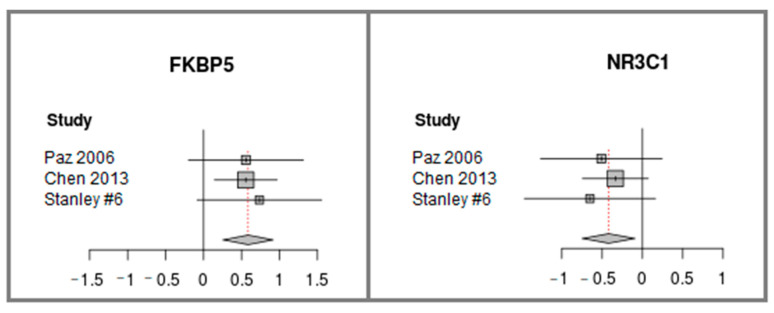
FKBP5 and NR3C1 differential expression Meta-analysis of three publicly available datasets of cerebellum samples of patients with schizophrenia vs. controls. The forest plot shows the differences in expression levels. It was generated using the function “forest” from the “meta” package in R, version 4.9-2 [41]. The datasets names appear along the *y*-axis. Each square represents the Effect size [42], the standardized difference between the expression in schizophrenia vs. controls, where the area of the square reflects the weight (proportional to the sample size) given to that dataset in the summary measure. The horizontal lines represent the 95% confidence interval for the Effect size of each dataset. The center of the diamond represents the summary of the overall Effect size across all studies, and its width represents the 95% confidence interval. The effect size direction is higher than zero if the expression is higher in schizophrenia vs. control.

Meta-analysis of FKBP and NR3C1 differential expression was computed using the three datasets (see Figure 3). While FKBP5 was found to be up-regulated in schizophrenia, NR3C1 was detected to be down-regulated. It should be noted that for most of the datasets, when analyzed separately, the two genes do not reach statistically significant differential expression (see the confidence intervals horizontal lines that cross the value of zero in Figure 3). Only when the meta-analysis of the datasets is performed, is statistical significance achieved. This demonstrates the power of the meta-analysis and its importance for producing statistically significant results. In addition, the consistency between the trends of the three datasets (towards FKBP5 up regulation and towards NR3C1 down-regulation (Figure 3)) increases the validity of the results. The raw expression patterns of FKBP5 and NR3C1 in each of the datasets are plotted in Supplementary Figures S2 and S3.

### 2.4. FKBP5 and NR3C1 Expression Patterns are Negatively Correlated

**Figure 4:**
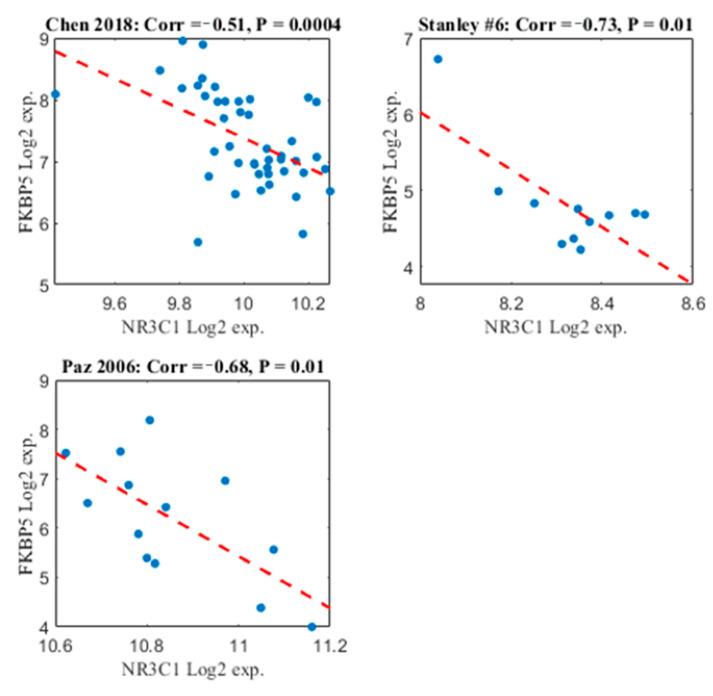
Scatter plot of FKBP5 and NR3C1 along patients with schizophrenia. The *x*-axis represents NR3C1 Log2 expression. The *y*-axis represents FKBP5 Log2 expression. Each point represents the Log2 expression of these two genes in a patient with schizophrenia, in a specific dataset. The dashed red line represents the linear regression line. Each of the three subplots represents each of the three datasets that were included in the meta-analysis. The Study name is written in the title, together with the Pearson correlation value and the *p*-value associated with the correlation value.

To further explore the validity of the results, we conducted correlation analysis between FKBP5 and NR3C1 expression. Pearson correlation was calculated in each of the datasets separately. The control samples were not included in this analysis in order to avoid “false positives,” as statistically significant negative correlation can be caused by the fact that FKBP5 is up-regulated while NR3C1 is down-regulated in the samples with schizophrenia vs. the controls, as was shown above. As shown in Figure 4, there is a significant negative correlation between the two genes in all cerebellum datasets that were included in the meta-analysis. This supports the results of the meta-analysis and reduces the likelihood of arbitrary findings.

### 2.5. Examination of Potential Confounding Factors

**Figure 5:**
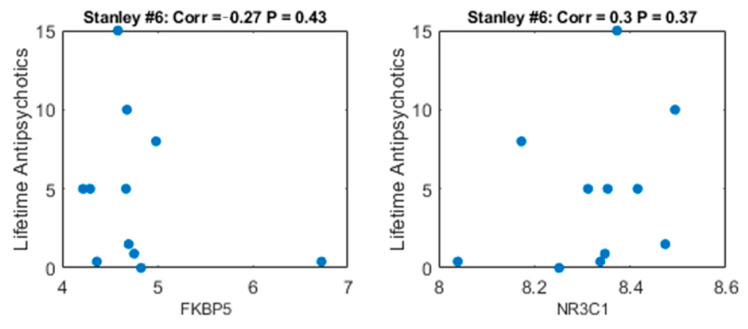
Scatter plot of lifetime quantity of antipsychotics (mg) vs. gene expression in Stanley #6 dataset. Left plot: FKBP5 gene. The *x*-axis represents Log2 expression, the *y*-axis represents Lifetime quantity of antipsychotics. Each point represents one of the individuals with schizophrenia. Right plot: the same for the NR3C1 gene.

A potential confounding factor that may underlie the differential expression we detect between the individuals with schizophrenia and the controls, is pharmacotherapy. To explore whether the differential expression of FKBP5 and NR3C1 was related to antipsychotics given to the patients, we applied Pearson correlation analysis between lifetime Fluphenazine or equivalent antipsychotics (in mg), available for the 11 Stanley#6 schizophrenia samples, and FKBP5 and NR3C1 expression levels. As shown in Figure 5, no significant correlation with antipsychotic treatment was measured. This suggests that the differential expression we detect of FKBP5 and NR3C1 is not caused by pharmacotherapy.

As described in Table 2, PMI values of the patients with schizophrenia were significantly higher than those of the healthy controls in the Chen 2013 study [40], which suggests it might act as a confounding factor. To further explore this, Pearson correlations were computed between PMI values and both FKBP5 and NR3C1 expression levels for the three datasets (Figures S4–S6). No significant correlation was measured between the PMI values and the expression of FKBP5 and NR3C1 in the three datasets. Thus, this analysis suggests that the differential expression we identify of both FKBP5 and NR3C1 is not the cause of differences in PMI values.

### 2.6. FKBP5 and NR3C1 are not Differentially Expressed in Blood Samples of Individuals with Schizophrenia Versus Healthy Controls

FKBP5 and NR3C1 differential expression was examined using a publicly available gene expression dataset of whole blood samples of 13 patients with schizophrenia and 8 controls [43]. The data was downloaded from the Gene Expression Omnibus database (GSE18312); see description of the dataset characteristics in the Supplementary Information. When standard two-sample *t*-test was applied, differential expression between the schizophrenia samples and the controls was not detected for FKBP5 (*p*-value = 0.92), nor for NR3C1 (*p*-value = 0.35); see also Figure S7.

### 2.7. FKBP5 and IL6 Expression Patterns are Positively Correlated

We performed an indirect exploration of the connection between FKBP5 expression and childhood trauma by conducting correlation analysis between FKBP5 and Interleukin 6 (IL6) expression. IL6 is a pro-inflammatory cytokine, whose hypo-methylation and up-regulation were associated with childhood trauma [44,45]. The results are presented in Figure S8. FKBP5 and IL6 show significant positive correlation of their expression patterns in the patients with schizophrenia in two out of the three datasets included in our meta-analysis. In the third dataset, Paz 2006 [39], there was a trend of positive correlation (*p*-value = 0.1).

## 3. Discussion

To the best of our knowledge, this is the first report of a systematic gene expression meta-analysis of FKBP5 and NR3C1 genes in postmortem cerebellum samples of individuals with schizophrenia (69 subjects) vs. healthy controls (78 subjects). We detect up-regulation of FKBP5 and down-regulation of NR3C1 in the cerebellum of the individuals with schizophrenia.

While for many years the cerebellum has been thought of as primarily dedicated to the coordination of motor activity, in recent years it has been shown to be involved in key cognitive functions [46]. Moreover, a reduction in cerebellar grey matter has been detected in individuals with schizophrenia [47] the degree of reduction is correlated with the severity of the positive symptoms of the patients [35,36]. Our decision to focus on this region was based on the evidence for its involvement in schizophrenia, and on the evidence that FKBP5 is highly expressed in the cerebellum, when compared to other brain regions (http://mouse.brain-map.org/; accessed at 26 February 2021) [37].

Our results are consistent with the FKBP5 gene-environment interaction model of schizophrenia, according to which childhood adversity leads to reduced DNA methylation at GREs of FKBP5, which results in up-regulation of FKBP5 and down-regulation of NR3C1, as we detect in the cerebellum of the patients. This model provides a molecular mechanism by which childhood adversity is involved in the development of schizophrenia. The negative correlation we detect between FKBP5 and NR3C1 expression levels is also consistent with the model and with previous findings [48], and increases the validity of the results. We did not have access to information regarding childhood adversity of the individuals that were included in our gene expression meta-analysis. However, a meta-analysis of prospective studies of 41,803 participants [2] found a significant association between childhood adversity, including trauma, and psychosis: the odds ratio was close to 3, pointing at a strong association between childhood adversity and psychosis, including schizophrenia. Thus, it would be plausible to assume that the individuals with schizophrenia had more exposure to childhood adversity than the healthy controls. In order to further explore this, we calculated the correlation between the expression levels of FKBP5 and IL6, a pro-inflammatory cytokine whose hypo-methylation and up-regulation were associated with childhood trauma [44,45] (see Figure S8). The significant positive correlation provides an indirect support to the association between childhood trauma and the up-regulation of both IL6 and FKBP5 in the patients with schizophrenia included in our meta-analysis. However, this necessitates further investigation.

FKBP5 expression in peripheral blood has been shown to serve as a potential biomarker in several psychiatric disorders. For example, it was found to be positively correlated with symptoms of anxiety and depression in women [49], was correlated with the response of individuals with PTSD to cognitive behavioral therapy [50] and with the response of individuals with depression to antidepressants [51]. While we did not detect differential expression of FKBP5 or NR3C1 in peripheral blood of patients with schizophrenia when compared to healthy controls (Figure S7), it should be noted that the analysis was performed on a single gene expression dataset (13 schizophrenia vs. 8 controls). Thus, additional work is needed to fully examine the potential of FKBP5 expression in peripheral blood as a biomarker in schizophrenia.

Increased FKBP5 expression in specific brain regions is associated with increased stress responsiveness and anxiety. Moreover, genetic knockout of FKBP5 in mice was shown to increase stress-coping behavior in the forced swim test [13,14,15] and pharmacological inhibition of FKBP5 was shown to improve stress-coping behaviors in mice [14,52]. The FKBP5 gene-environment interaction model has been shown to be related to various psychiatric disorders [6]. Similarly to our findings in schizophrenia, FKBP5 was shown to be up-regulated in post mortem brain samples of patients with PTSD [11], major depressive [53] and bipolar [54] disorders. Thus, our findings in schizophrenia are similar to previous findings of other psychiatric disorders in which this model was shown to be involved. 

Our study, like other postmortem studies, has several limitations. As postmortem studies represent only a snapshot of neurobiology at the end of life, they cannot directly address the abnormalities that may have existed when the disorder was first expressed. This is highly important for schizophrenia, as there is evidence that its pathogenesis is rooted in early development [55]. The fact that the three datasets that were included in the meta-analysis consist of relatively young patients (mean age 45) overcomes this limitation only partially. In addition, antipsychotic treatment has the potential to affect gene expression. This limitation was partially addressed by the correlation analysis we performed, which found no significant association between antipsychotic treatment and the expression of both FKBP5 and NR3C1 in the Stanley #6 dataset. When we examined the potential effect of PMI, no significant correlation was detected between PMI values and the genes’ expression in the three datasets (Figures S4–S6). Moreover, the integration of independent datasets and the significant negative correlation between the expression of FKBP5 and NR3C1 increase the robustness and reliability of the results. As gene expression does not always correlate with the levels of the proteins coded by the genes, the fact that we measure gene expression alone is a serious limitation, which causes difficulties in making definitive conclusions regarding the biological consequences of the signal we detect. In this context, it should be mentioned that while FKBP5 was found to be up-regulated in prefrontal cortex of patients with schizophrenia in [48], no change in its protein level was detected [48]. Additional study is needed to decipher the consequences of the differential expression we identify in schizophrenia, in terms of protein levels and functionality. Another limitation is the different patterns of gene expression in distinct brain regions, as was shown in [33] for healthy individuals. Interestingly, the cerebellum expression patterns differed the most from the other brain regions [33]. Previous schizophrenia FKBP5 expression studies showed up-regulation in a subgroup of the brain regions, while others showed no differential expression (summarized in Table 1). Moreover, brain region was found as an influential factor on FKBP5 expression (*p*-value < 0.07) in [26]. These findings suggest that different brain regions should be studied separately. We thus analyzed cerebellum samples only, without including additional regions.

In summary, in a systematic meta-analysis we detect up-regulation of FKBP5 and down-regulation of NR3C1 in cerebellum samples of individuals with schizophrenia, and negative correlation between their expression patterns. These findings support the FKBP5 gene-environment interaction model in schizophrenia, suggesting a molecular mechanism by which childhood adversity is involved in its development. While FKBP5 expression in peripheral blood was proposed as a potential biomarker of psychiatric disorders, we did not find differential expression in blood samples of patients with schizophrenia vs. healthy controls. However, this needs further investigation. To explore FKBP5 potential as a therapeutic target, a mapping of its differential expression patterns along different brain regions in schizophrenia is needed. Moreover, further study is needed to decipher the consequences of the differential expression we identify, in terms of protein levels and functionality.

## 4. Materials and Methods

### 4.1. Eligible Gene Expression Datasets Selection for Meta-analysis

Publicly available gene expression datasets were searched in four public repositories: NCBI Gene Expression Omnibus (GEO) (http://www.ncbi.nlm.nih.gov/geo/; accessed at 12 May 2020), the Stanley Medical Research Institute (SMRI) Array Collection (http://www.stanleyresearch.org/brain-research/array-collection/; accessed at 10 April 2020) and the CommonMind (CMC) [55] and psychENCODE [56] consortiums of the National Institute of Mental Health (NIMH). The key words that were used were: schizophrenia, cerebellum, gene expression, human, brain samples. Figure 1 presents the workflow of eligible dataset selection, following the PRISMA 2009 guidelines [34].

The following information was extracted for each dataset: platform, number of patients and healthy individuals, gender, descriptive statistics of age, pH and post mortem interval (PMI), when available (Table 2), and the preprocessed gene expression data. Inclusion criteria that were used for dataset selection were: human schizophrenia versus healthy individuals, post mortem cerebellum samples, a minimum of seven samples of individuals with schizophrenia, comparable conditions and availability of gene expression preprocessed data. The full datasets characteristics are described in the Supplementary Information.

### 4.2. Gene Expression Meta-analysis

For a given gene, a meta-analysis that integrates its expression in the cerebellum datasets was computed as follows. Effect size (Hedges’ g [57]), the standardized difference between the expression in the schizophrenia vs. healthy control samples, was computed independently for each of the datasets. The effect size was above zero if the expression in the schizophrenia samples was higher than in the controls. Hedges’g and confidence interval values were calculated for each dataset using the function “metacont” of the “meta” package in R, a general package for meta-analysis, version 4.9-2 [42]. The summary measure with its confidence interval was calculated by the same function, with the use of the random effects model [38].

## Figures and Tables

**Table 1:** Literature review of postmortem brain studies of FKBP5 differential expression in schizophrenia vs. healthy controls. Cohorts that are marked in bold overlap between the studies. #, number of; SZ, schizophrenia; CNT, controls; BA, Brodmann area; STG, superior temporal gyrus; DLPFC, dorsolateral prefrontal cortex.

Study	# Subjects with Schizophrenia and Controls per Region and Cohort	Method	FKBP5 Differential Expression
(Darby et al., 2016) [24]	Hippocampus: SZ (35), CNT (32);	RNA sequencing;	Up-regulation;
	Orbitofrontal cortex: SZ (13), CNT (15);	RNA sequencing;	No differential expression
(Sinclair, Fillman, Webster, & Weickert, 2013) [30]	Prefrontal cortex: SZ (20), CNT (20);	RNA sequencing;	Up-regulation;
	Prefrontal cortex: SZ (35), CNT (35)	qPCR	Up-regulation
(Gandal et al. 2018) Meta-analysis	**BA46: SZ (35), CNT (34);** **BA46: SZ (30), CNT (29);** **BA46: SZ (15), CNT (19);** **BA10: SZ (28), CNT (23);** Parietal cortex: SZ(51), CNT (50); **Total:** SZ (159), CNT (155)	Microarray	Up-regulation (union of the datasets)
(Jiang et al. 2017 Meta-analysis [26]	**BA46: SZ (35), CNT (34);**	Microarray	No differential expression (union of the datasets)
	**BA46: SZ (30), CNT (29);**		
	**BA46: SZ (15), CNT (19);**		
	**BA10: SZ (28), CNT (23);** BA10: SZ (13), CNT (15); Hippocampus: SZ (15), CNT (18); Striatum: SZ (18), CNT (18); Entorhinal cortex: SZ (10), CNT (10); STG: SZ (23), CNT (19); STG: SZ (9), CNT (9); STG: SZ (8), CNT (8); DLPFC: SZ (65), CNT (72); **Total:** SZ(196), CNT (72)		
SZDB2.0 database (Wu et al., 2020, 2017), using (Fromer et al., 2016) CMC data [27,28,29]	DLPFC: SZ (258), CNT (279);	RNA sequencing	No differential expression

**Table 2:** **Characteristics of individual studies included in the meta-analysis.** Abbreviations: #: number of; SZ: schizophrenia; CNT: controls; PMI: post mortem interval; M: males; F: females.

Study	Accession	# SZ	# CNT	Platform	Mean Age (Standard Deviation	Mean PMI (Standard Deviation	Mean pH (Standard Deviation)
Paz 2006 [39]	GDS1917	14,14M:0F	14,14M:0F	U133 Plus 2.0 Array	SZ: 45 (12)	SZ: 15.6 (6)	Not provided
					CNT: 43 (10)	CNT: 12.2 (5)	
					*p* = 0.57	*p* = 0.11	
Chen 2013 [40]	GSE35978	44,33M:12F	50,31M:19F	Gene 1.0 ST Array	SZ: 46 (9)	SZ: 33 (15)	SZ: 6.5 (0.3)
					CNT: 43 (9) *p* = 0.2	CNT: 28 (11) *p* = 0.042	CNT: 6.4 (0.3) *p* = 0.44
	Stanley#6	11, 8M:3F	14,9M:5F	U95 Av2 Array	SZ: 47 (9)	SZ: 25 (10)	SZ: 6.3 (0.3)
					CNT: 46 (14)	CNT: 34 (14)	CNT: 6.2 (0.3)
					*p* = 0.83	*p* = 0.071	*p* = 0.82
						
		Total: 69	Total: 78				
						

## Data Availability

Publicly available datasets were analyzed in this study (listed in Table 2, with accession numbers). The datasets can be found in the GEO (http://www.ncbi.nlm.nih.gov/geo/; accessed at 22 February 2021) and the SMRI Array Collection (http://www.stanleyresearch.org/brain-research/array-collection/; accessed at 22 February 2021) databases.

## Supplementary Materials

The following are available online at https://www.mdpi.com/2075-1729/11/3/190/s1, Figure S1: Box plot of FKBP5 schizophrenia vs. controls Log2 expression of the CommonMind Consortium data (Fromer et al., 2016) composed of 258 DLPFC samples of patients with schizophrenia vs. 279 healthy controls, Figure S2. FKBP5 Log2 expression in the three cerebellum datasets, Figure S3. NR3C1 Log2 expression in the three cerebellum datasets, Figure S4. Scatter plot of PMI vs. gene expression, along the 44 Chen 2013 dataset (GSE35978) individuals with schizophrenia, Figure S5. Scatter plot of PMI vs. gene expression, along the 14 Paz 2006 dataset individuals with schizophrenia, Figure S6. Scatter plot of PMI vs. gene expression, along the 11 Stanley#6 dataset individuals with schizophrenia, Figure S7. FKBP5 and NR3C1 Log2 expression in Bousman 2010 dataset (GSE18312), Figure S8 Scatter plot of FKBP5 and IL6 along patients with schizophrenia. The x-axis represents IL6 Log2 expression.
