# Supplementary material for: Gene Expression Meta-Analysis of Cerebellum Samples Supports the FKBP5 Gene-Environment Interaction Model for Schizophrenia
Source: Life (Basel). 2021 Feb 27;11(3):190. doi: 10.3390/life11030190 (PMC7997256; doi:10.3390/life11030190)
Supplement: Supplementary file 1 [file life-11-00190-s001.pdf]

life

Article

Gene Expression Meta-analysis of Cerebellum Samples Supports the FKBP5 Gene-environment Interaction Model for Schizophrenia

Libi Hertzberg [1,2,*], Ada H Zohar [3], Assif Yitzhaky [1]

[1] Department of Physics of Complex Systems, Weizmann Institute of Science, 76100 Rehovot, Israel; assif.yitzhaky@weizmann.ac.il
[2] Shalvata Mental Health Center, affiliated with the Sackler School of Medicine, Tel-Aviv University, 69978 Tel Aviv, Israel
[3] Department of Behavioral Sciences, Ruppin Academic Center, 40250 Hefer Valley, Israel; adaz@ruppin.ac.il
* Correspondence: libi.hertzberg@gmail.com; Tel.: +972-9-7478554; Fax: +972-9-7478923

Abstract: Background: One of the most studied molecular models of gene-environment interactions is that of FKBP5, which has been shown to interact with childhood adversity to increase the risk of psychiatric disorders, and has been implicated in schizophrenia. While the model predicts up-regulation of FKBP5, previous brain samples gene expression studies yielded inconsistent results. Methods: We performed a systematic gene expression meta-analysis of FKBP5 and NR3C1, a glucocorticoid receptor inhibited by FKBP5, in cerebellum samples of patients with schizophrenia. The gene expression databases GEO, SMRI and those of NIMH were searched, and out of six screened datasets, three were eligible for the meta-analysis (overall 69 with schizophrenia and 78 controls). Results: We detected up-regulation of FKBP5 and down-regulation of NR3C1 in schizophrenia, and a negative correlation between their expression patterns. Correlation analysis suggested that the detected differential expression did not result from potential confounding factors. Conclusions: Our results give significant support to the FKBP5 gene-environment interaction model for schizophrenia, which provides a molecular mechanism by which childhood adversity is involved in the development of the disorder. To explore FKBP5's potential as a therapeutic target, a mapping of its differential expression patterns in different brain regions of schizophrenia patients is needed.

Keywords: gene expression; meta-analysis; schizophrenia; FKBP5; cerebellum

Citation: Hertzberg, L.; Zohar, A.H.; Yitzhaky, A. Gene Expression Meta-Analysis of Cerebellum Samples Supports the FKBP5 Gene-Environment Interaction Model for Schizophrenia. *Life* **2021**, *11*, 190. https://doi.org/10.3390/life11030190

Received: 1 February 2021
Accepted: 22 February 2021
Published: 27 February 2021

Publisher's Note: MDPI stays neutral with regard to jurisdictional claims in published maps and institutional affiliations.

Copyright: © 2021 by the authors. Submitted for possible open access publication under the terms and conditions of the Creative Commons Attribution (CC BY) license (http://creativecommons.org/licenses/by/4.0/).

Supplementary Materials:

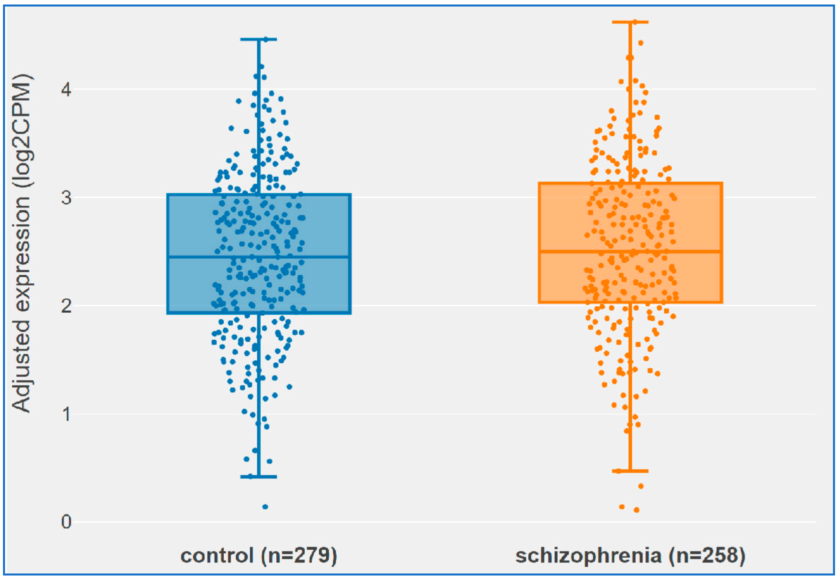

Figure S1. Box plot of FKBP5 schizophrenia vs. controls Log2 expression of the CommonMind Consortium data (Fromer *et al.*, 2016) composed of 258 DLPFC samples of patients with schizophrenia vs. 279 healthy controls. The figure was constructed using the SZDB2.0 database (Wu, Yao and Luo, 2017; Wu et al., 2020) (http://www.szdb.org/). The y–axis corresponds to the Log2 counts per million (CPM) expression value. On each box, the central mark (horizontal line) indicates the median, and the bottom and top edges of the box indicate the 25th percentile and 75th percentile, respectively. The Boxplot is plotted on top of the raw data samples, where each point represents the Log2 CPM of a specific sample. The p-value for having differential expression between schizophrenia and controls was calculated to be 0.55.

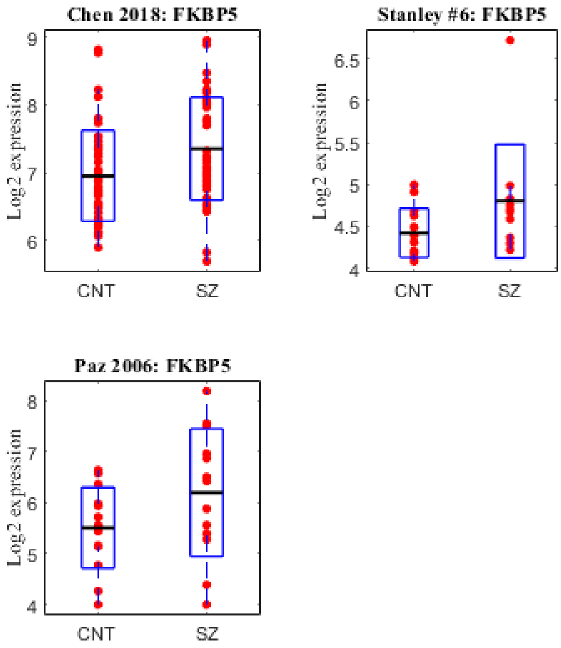

Figure S2. FKBP5 Log2 expression in the three cerebellum datasets. Log2 expression is plotted for each of the two sample groups separately (healthy controls, labeled "CNT" and patients with schizophrenia, labeled "SZ"). The y-axis represents the Log2 expression. On each box, the central mark (horizontal black line) indicates the mean, and the bottom and top edges of the blue box indicate one standard deviation. The Boxplot is plotted on top of the raw data samples, where each red point represents the Log2 expression of a specific sample. Each of the 3 subplots represents

each of the 3 datasets that were included in the meta-analysis. The Study name is specified in the title.

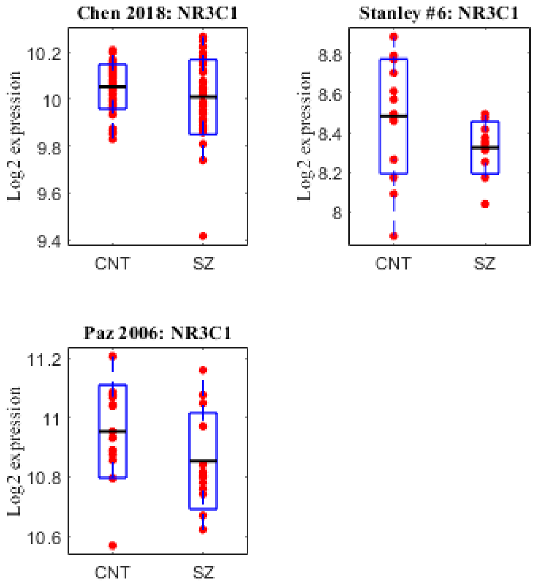

Figure S3. NR3C1 Log2 expression in the three cerebellum datasets. Log2 expression is plotted for each of the two sample groups separately (healthy controls, labeled "CNT" and patients with schizophrenia, labeled "SZ"). The y-axis represents the Log2 expression. On each box, the central mark (horizontal black line) indicates the mean, and the bottom and top edges of the blue box indicate one standard deviation. The Boxplot is plotted on top of the raw data samples, where each red point represents the Log2 expression of a specific sample. Each of the 3 subplots represents each of the 3 datasets that were included in the meta-analysis. The Study name is specified in the title.

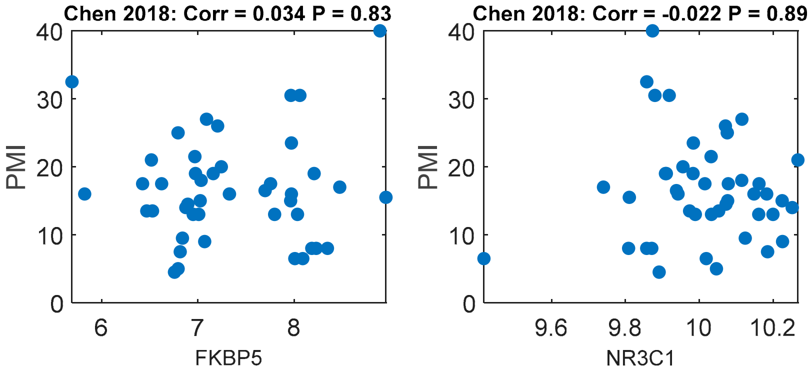

Figure S4. Scatter plot of PMI vs. gene expression, along the 44 Chen 2013 dataset (35) (GSE35978) individuals with schizophrenia. For FKBP5 (left plot) and NR3C1 (right plot), the X-axis represents Log2 expression, the y-axis represents PMI. Each point represents one of the 44 individuals with schizophrenia. Pearson correlation values and the associated p-values are written in the title of each plot**.**

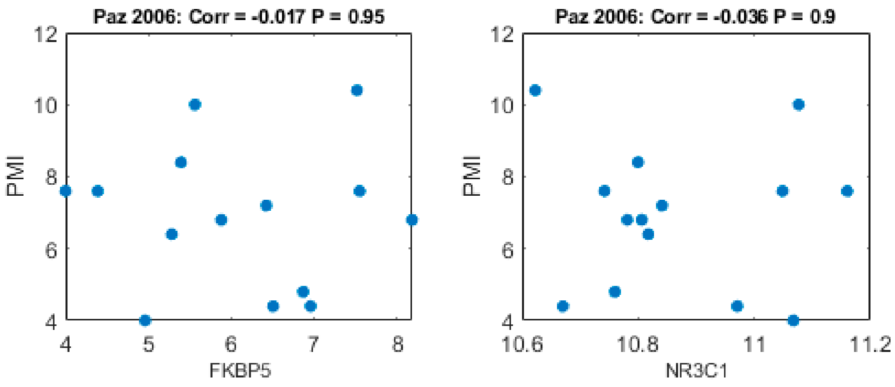

Figure S5. Scatter plot of PMI vs. gene expression, along the 14 Paz 2006 dataset individuals with schizophrenia. For FKBP5 (left plot) and NR3C1 (right plot), the X-axis represents Log2 expression, the y-axis represents PMI. Each point represents one of the 14 individuals with schizophrenia. Pearson correlation values and the associated p-values are written in the title of each plot.

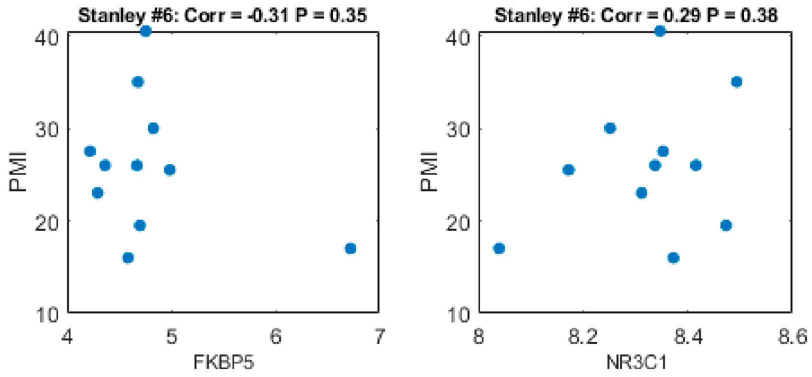

Figure S6. Scatter plot of PMI vs. gene expression, along the 11 Stanley#6 dataset individuals with schizophrenia. For FKBP5 (left plot) and NR3C1 (right plot), the X-axis represents Log2 expression, the y-axis represents PMI. Each point represents one of the 11 individuals with schizophrenia. Pearson correlation values and the associated p-values are written in the title of each plot.

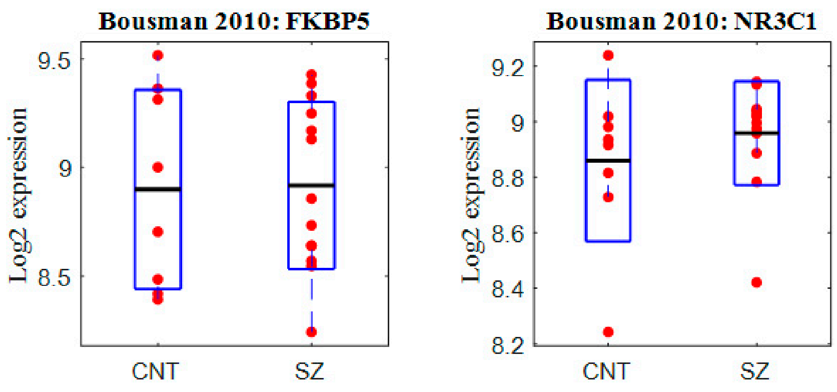

Figure S7. FKBP5 and NR3C1 Log2 expression in Bousman 2010 dataset (3) (GSE18312). Log2 expression is plotted for each of the two sample groups separately (healthy controls, labeled "CNT" and patients with schizophrenia, labeled "SZ"). The y-axis represents the Log2 expression. On each blue box, the central mark (horizontal black line) indicates the mean, and the bottom and top edges of the box indicate one standard deviation. The Boxplot is plotted on top of the raw data samples, where each red point represents the Log2 expression of a specific sample. FKBP5 Log2 expression is plotted on the left plot and NR3C1 is plotted on the right plot.

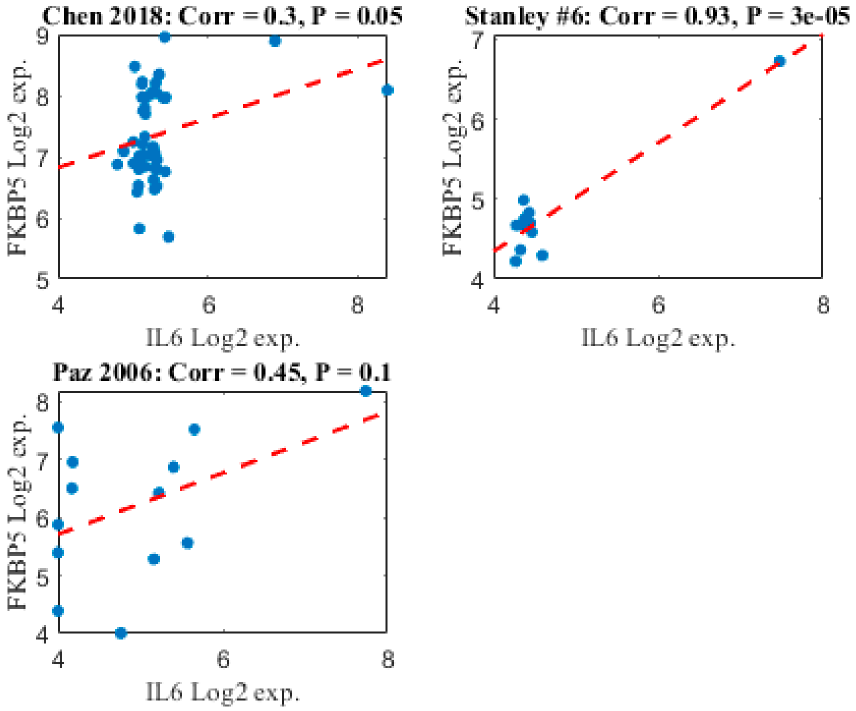

Figure S8 Scatter plot of FKBP5 and IL6 along patients with schizophrenia. The x-axis represents IL6 Log2 expression. The y-axis represents FKBP5 Log2 expression. Each point represents the Log2 expression of these two genes in a patient with schizophrenia, in a specific dataset. The dashed red line represents the linear regression line. Each of the 3 subplots represents each of the 3 datasets that were included in the meta-analysis. The Study name is written in the title, together with the Pearson correlation value and the p-value associated with the correlation value.

Cerebellum datasets characteristics

Paz et al. 2006 (1), GEO accession: GDS1917

The dataset was downloaded from the GEO database (https://www.ncbi.nlm.nih.gov/sites/GDSbrowser?acc=GDS1917).The initial dataset consists of 28 brain samples corresponding to the crus I/VIIa area of the cerebellum from subjects with schizophrenia (n=14) and healthy controls (n=14). (The samples were obtained from the Maryland Brain Collection). The samples were run on Affymetrix Human Genome U133 Plus 2.0 Array. Normalization method: MAS5.0. We applied threshold and Log2 transformation. The threshold value was determined using scatter plots of healthy control samples in order to estimate the noise level (the threshold after Log2 that was used is 4). Filtering: The initial number of probe-sets was 54,613. 1) In case of a gene symbol with multiple probe-sets, the probe-set with the highest mean expression over the samples was taken into account and the other probe-sets were discarded. Number of genes after this step: 30,803. 2) Genes that were absent (values equal or lower than the threshold) in more than 70% of both the schizophrenia and the control samples, were filtered out. Number of genes after filtering: 27,329

Chen et al. 2013 (4), GEO accession: GSE35978

The dataset was downloaded from the GEO database (https://www.ncbi.nlm.nih.gov/geo/query/acc.cgi?acc=GSE35978). The initial dataset consisted of 312 brain samples, including subjects with schizophrenia (n=44) and healthy controls (n=50) from the cerebellum. Samples were run on Affymetrix Human Gene 1.0 ST Array (transcript (gene) version). Normalization method: "The data were analyzed by RMA using Affymetrix Expression Console with default analysis settings"

(described in GSE35978_series_matrix.txt, available at the aforementioned link). After RMA (Robust Multiarray Average) normalization, no threshold or log2 are needed, as it includes the following steps: 1) Background correction 2) Quantile normalization 3) Log2 transformation. Filtering: The initial number of probe-sets was 33,297 (25,293 with gene symbols). In case of a gene symbol with multiple probe-sets, the probe-set with the highest mean expression over the samples was taken into account and the other probe-sets were discarded. Number of genes after this step: 23,307.

Stanley Study ID 6 from the Stanley Medical Research Institute

The dataset was downloaded from The Stanley Online Genomics Database (https://www.stanleygenomics.org/). Investigator: Feinberg. The dataset consists of 25 cerebellum samples from subjects with schizophrenia (n=11) and healthy controls (n=14). Array type: Affymetrix hgu95av2. Normalization method: RMA. After applying RMA, no threshold or Log2 transformation are needed, as was described above. Filtering: The initial number of probe-sets was 12,159. In case of a gene symbol with multiple probe-sets, the probe-set with the highest mean expression over the samples was taken into account and the other probe-sets were discarded. Number of genes after this step: 9,081.

Blood samples dataset characteristics

Bousman et al. 2010 (42), GEO accession: GSE18312

The dataset was downloaded from the GEO database (https://www.ncbi.nlm.nih.gov/geo/query/acc.cgi?acc=GSE18312). The original dataset consisted of 30 blood samples, including subjects with schizophrenia (n=13) and healthy controls (n=8). Platform: Affymetrix Human Exon 1.0 ST Array [transcript (gene) version]. Normalization method: "The data were analyzed with Partek Genomics Suite v6.4 using RMA to analyze probe intensities and to determine differential gene expression between diagnostic and control groups" (described in GSE18312_series_matrix.txt, available at the aforementioned link). Filtering: The initial number of gene symbols was 17,634. In case of a gene symbol with multiple probe-sets, the probe-set with the highest mean expression over the samples was taken into account and the other probe-sets were discarded. Number of genes after this step: 17,324.

References

1. Paz RD, Andreasen NC, Daoud SZ, Conley R, Roberts R, Bustillo J, Perrone-Bizzozero NI (2006): Increased Expression of Activity-Dependent Genes in Cerebellar Glutamatergic Neurons of Patients With Schizophrenia. *Am J Psychiatry* 163: 1829–1831.
2. Oldham MC, Konopka G, Iwamoto K, Langfelder P, Kato T, Horvath S, Geschwind DH (2008): Functional organization of the transcriptome in human brain. *Nat Neurosci* 11: 1271–1282.
3. Bousman CA, Chana G, Glatt SJ, Chandler SD, Lucero GR, Tatro E, *et al.* (2010): Preliminary evidence of ubiquitin proteasome system dysregulation in schizophrenia and bipolar disorder: convergent pathway analysis findings from two independent samples. *Am J Med Genet B Neuropsychiatr Genet* 153B: 494–502.
4. Chen C, Cheng L, Grennan K, Pibiri F, Zhang C, Badner JA, *et al.* (2013): Two gene co-expression modules differentiate psychotics and controls. *Mol Psychiatry* 18: 1308–1314.
5. Mudge J, Miller NA, Khrebtukova I, Lindquist IE, May GD, Huntley JJ, *et al.* (2008): Genomic convergence analysis of schizoprenia: mRNA sequencing reveals altered synaptic vesicular transport in post-mortem cerebellum. *PLoS One* 3. https://doi.org/10.1371/journal.pone.0003625
